# Effect of center of rotation of angulation-based levelling osteotomy on instantaneous center of rotation ex vivo

**DOI:** 10.1007/s11259-024-10314-2

**Published:** 2024-01-29

**Authors:** James Edward Miles, Parisa Mazdarani

**Affiliations:** 1 Department of Veterinary Clinical Sciences, University of Copenhagen, Dyrlaegevej 16, Frederiksberg C, 1870 Denmark; 2 Current address: College of Veterinary Medicine, University of Florida, 2015 SW 16th Ave, Gainesville, FL 32608 USA

**Keywords:** Dogs, Biomechanics, Cranial cruciate ligament rupture, Stifle joint, Kinematics, Percentage gliding

## Abstract

Cranial cruciate ligament rupture is a common cause of femorotibial instability in dogs. Despite numerous techniques being described for achieving joint stabilization, no consensus exists on the optimal management strategy. This ex vivo study utilized the path of the instantaneous center of rotation (ICR) to compare normal, pathological and treated joints. Fluoroscopic recordings of seven limbs from a previous study of canine stifle joint stability following center of rotation of angulation-based levelling osteotomy (CBLO) with and without hamstring loading were analyzed using least-squares approximation of the ICR and estimation of percentage gliding (vs. rolling) to determine if alterations in ICR path and gliding caused by CCL transection and following meniscal release could be normalized by CBLO. In intact joints, the ICR path was located mid-condyle, but this shifted significantly proximally and caudally following CCL transection and medial meniscal release (*p* < 0.007, *p* < 0.04). Hamstring loading resulted in qualitative and some quantitative improvements in joint movement based on percentage gliding movement analysis. The ICR path after CBLO remained significantly different to the intact location with or without a hamstring load (*p* < 0.02, *p* < 0.04), potentially consistent with CBLO aims of mild residual instability. CBLO resulted in percentage gliding characteristics not significantly different to intact joints (*p* > 0.08). Qualitative improvements in ICR path and percentage gliding quantities and variability suggest that hamstring loading positively influences joint biomechanics and that further investigation of this role ex vivo and clinically is warranted.

**Supplementary Information:**

The online version contains supplementary material available at 10.1007/s11259-024-10314-2.

## Introduction

Cranial cruciate ligament (CCL) rupture is a common cause of canine stifle disability (Engdahl et al. 2021), and the choice of technique to stabilize affected joints remains controversial (von Pfeil et al. 2018). The center of rotation of angulation (CORA)-based levelling osteotomy (CBLO) is a recent addition to stabilization surgeries which has shown promise clinically (Vasquez et al. 2018) and in ex vivo and in silico studies (Mazdarani et al. 2022; Putame et al. 2019).

Recently, the instantaneous center of rotation (ICR) path was used to assess the triple tibial osteotomy procedure (Mazdarani et al. 2023). This path varies with the amount of rolling or gliding at the joint surfaces as well as the presence or absence of joint pathology (Hollman et al. 2002; Ireland et al. 1986; Mazdarani et al. 2023; Mitton et al. 1991).The majority of movement in the stifle joint is about the mediolateral axis, at least in intact stifles (Korvick et al. 1994; Tinga et al. 2018), allowing movement to be treated as approximately planar (Soudan et al. 1979). While issues exist with the use of ICR paths, such as dependency on start and end positions, the angular change between positions, landmark placement, and calculation methods (Challis 2001; Chen and Katona 1999), interpolation methods and fitting with least-squares can address many of these (Mazdarani et al. 2023).

Weight-bearing influences ICR path in humans (Hollman et al. 2002), and variations in simulated muscle loading affect ex vivo canine joint stability before and after CBLO (Mazdarani et al. 2022): in the latter study simulated quadriceps and gastrocnemius loads of 147 N and 49 N, respectively, were tested alone or in combination with a hamstring load of 29 N. Hamstring loading resulted in improved joint stability in this study. Given in vivo evidence of better medium- to long-term joint health following CBLO (Vasquez et al. 2018) compared to tibial plateau leveling osteotomy (Hulse et al. 2010), it seems valid to consider whether CBLO results in a distinct ICR path compared to other osteotomies.

We hypothesized that CBLO would result in partial normalization of the ICR path in the CCL deficient stifle joint ex vivo under constant quadriceps and gastrocnemius loads, and that normalization would be positively affected by addition of a hamstring load to the model.

## Methods

### Source material

Coordinate data were obtained from an ex vivo fluoroscopic study of canine stifle joint stability (Mazdarani et al. 2022) and a similar analytical approach to that previously described was employed (Mazdarani et al. 2023). Briefly, seven left hind limbs were instrumented, including placement of fiduciary markers at the origin and insertion of the CCL, and in the distal femoral and proximal tibial diaphysis (3 each) to indicate the bone axes. Limbs were mounted on a custom frame with simulated quadriceps and gastrocnemius loads (147 N and 49 N, respectively) and an optional hamstring load of 29 N created using lines connected to the patella, calcaneus, and proximal tibial metaphysis, respectively, and passing around pulleys to align the loads with muscle lines of action. Simulated muscle loads were based on previous biomechanical studies and experimental results (Jensen et al. 2020; Mazdarani et al. 2022; Shahar and Banks-Sills 2004). No compressive load to simulate weight-bearing was applied. Limbs were aligned parallel to and 10 cm (± 0.5 cm) from the image receptor. Fluoroscopic recordings were obtained at 25 frames per second during gradual movement from flexion to extension for intact stifles, following CCL transection (CCLx), then medial meniscal release (MMR), and finally following CBLO to a target tibial plateau angle of 10°. Still images were extracted, and landmarks and markers identified by a single observer to yield coordinate data through the range of motion. The length of Blumensaat’s line (Blumensaat 1938) was used to define a scale between limbs and recordings by setting this length to 100%, and a coordinate system was defined from the cranial aspect of this line (0,0) to the caudal aspect (0,100). All output data were then rotated and scaled according to this so that Blumensaat’s line became the x-axis using custom macros in Visual Basic for Applications (Miles 2022).

### Estimation of the ICR

Five key tibial landmarks (tibial tuberosity, cranioproximal tibia, CCL insertion marker, caudal tibial plateau, and proximal tibial diaphyseal marker) were used to calculate ICR position at 60° overlapping steps from 55° to 135° using a least squares approach (Challis 2001; Mazdarani et al. 2023), giving five locations for each limb and condition: locations were identified by the mid- movement angle prefixed by “m”, e.g. m85° for the movement 55° to 115°. Variability was quantified using 67% error ellipses (Sokal and Rohlf 2012).

### Femoral condylar surface approximation

For each still image, the femorotibial contact point (FTCP) was identified, defined as the point on the femoral condylar surface closest to the tibial plateau at any given joint angle for each limb. Best-fit circles for the femoral condylar surface of the intact stifles were calculated using Taubin’s method (Fiedler 2018), and all FTCP coordinates were scaled, interpolated and smoothed to these circles.

### Joint movement characteristics

Joint movement was characterized using percentage gliding (Hollman et al. 2002). The arc of the femoral condylar surface $${S}_{m}$$ for the angular difference $$\theta$$ between coordinate pairs was calculated using the radius of the best-fit circle $${r}_{bf}$$, where $${S_m} = {r_{bf}} \cdot \theta$$. The ICR radius $${r}_{icr}$$was defined as the distance from the ICR to the FTCP location at mid-angular difference, and the arc $${S}_{f}$$ calculated as $${S_f} = \left( {{r_{bf}} - {r_{icr}}} \right) \cdot \theta$$. Percentage rolling (PR) was calculated as $$PR = 100 \cdot {\raise0.7ex\hbox{${{S_m}}$} \!\mathord{\left/{\vphantom {{{S_m}} {\left( {{S_m} + \left( {{S_m} - {S_f}} \right)} \right)}}}\right.\kern-\nulldelimiterspace}\!\lower0.7ex\hbox{${\left( {{S_m} + \left( {{S_m} - {S_f}} \right)} \right)}$}}$$, and percentage gliding as $$100-PR$$ (Supplemental Fig. 1).

### Statistics

Analysis was performed using SPSS 29.0 for Windows (IBM Statistics, Armonk, NY). Univariate normality was assessed using the Shapiro-Wilk test. Multivariate outliers were identified using the Mahalanobis distance and critical chi-squared values for 2 degrees of freedom. One-way multivariate ANOVA with pairwise multivariate post hoc comparisons from the DISCRIMINANT procedure were used to compare the x and y coordinates for ICR locations within or between joint stability situations. Results were reported using Pillai’s trace test statistic (V) and the approximate F-statistic with degrees of freedom as F(df), and effect size was estimated using partial omega-squared (${\omega }_{p}^{2}$) and cut-offs between small, medium, and large effects of 0.13 and 0.26 (Tomczak and Tomczak 2014). Percentage gliding was analyzed using Friedman’s ANOVA to compare between stability situations or between joint angles. Results were reported using the ${\text{X}}^{2}$ statistic with degrees of freedom in parentheses and effect size was estimated using Kendall’s W with cut-offs between small, medium, and large effects of 0.3 and 0.5 (Tomczak and Tomczak 2014). Significance was set at *p* ≤ 0.05.

## Results

Mean body mass of the cadavers was 31.7 kg (SD 2.4 kg) and mean tibial plateau angles before and after CBLO were 28.1° (SD 4.3°) and 9.7° (SD 2.3°). A previous analysis using the same limbs as the current study found that at a standing joint angle (135°) following CBLO, 3/7 stifles were stable without a hamstring load, and 5/7 with (Mazdarani et al. 2022).

### Multivariate outliers

Under zero hamstring load, 2, 3, and 1 ICR x,y coordinate pairs for CCLx, MMR and CBLO conditions, respectively, were identified as multivariate outliers. With hamstring load, 1, 2 and 1 coordinate pairs for CCLx, MMR and CBLO conditions were outliers. MANOVA was performed with and without outlier removal.

### ICR location

**Fig. 1:**
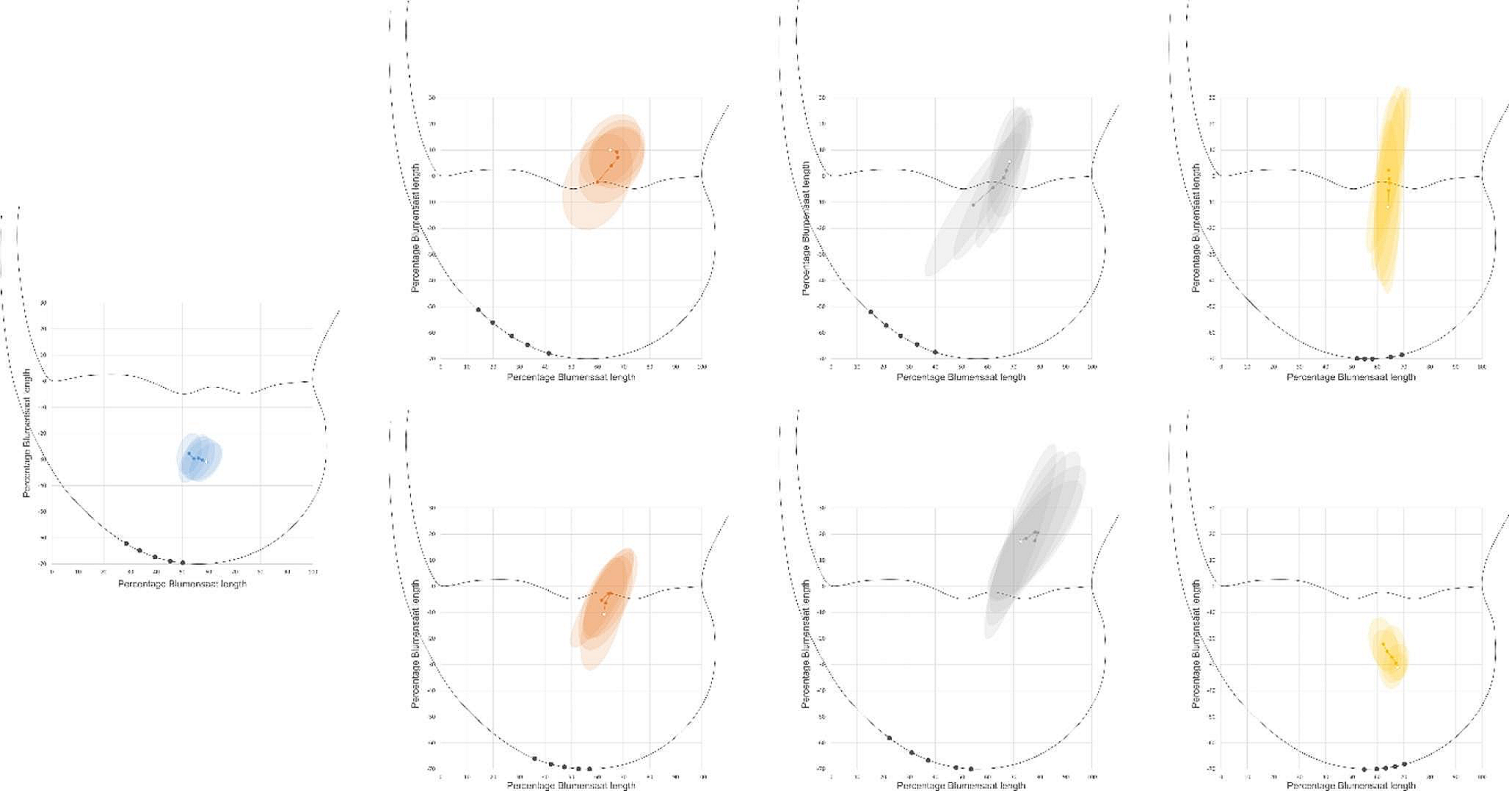
Location of the instantaneous center of rotation (ICR) under varying joint angles and joint stability locations. Locations were scaled between limbs by using the length and orientation of Blumensaat’s line, giving this a value of 0-100 along the x-axis and corresponding values along the y-axis. From left to right: intact, cranial cruciate ligament transection (top – without hamstring load, bottom – with), medial meniscal release (top- without, bottom – with), and CORA-based leveling osteotomy (top – without, bottom – with). The white marker indicates the start of the ICR path at flexion, and the shaded areas represent 67% error ellipses. The markers along the condylar outline indicate approximate positions of the femorotibial contact point used for determining percentage gliding

Location of the ICR varied with caudal joint angle for intact, CCLx without and with hamstring load, and CBLO with hamstring load (intact: V = 0.99, F(8) = 5.9, *p* < 0.001, ${\omega }_{p}^{2}$=0.41; CCLx without: V=1.32, F(8)=7.7, *p*<0.001, ${\omega }_{p}^{2}$=0.57; CCLx with: V=1.02, F(8)=6.3, *p*<0.001, ${\omega }_{p}^{2}$=0.43; CBLO with: V=0.92, F(8)=4.3, *p* < 0.001, ${\omega }_{p}^{2}$=0.35), but not for any other condition (Fig. 1; Supplementary Table 1).

Without a hamstring load, intact ICR locations differed from CCLx, MMR and CBLO ICR locations at all joint angles, and apart from CCLx vs. CBLO at m85°, no other pairwise differences were observed (Table 1). With the addition of a hamstring load, multivariate pairwise differences were observed for all comparisons and all joint angles. With few exceptions, inclusion of multivariate outliers did not affect this overall pattern (Supplementary Table 2).

**Table 1:** Effect of joint stability condition on instantaneous center of rotation location at tested joint angles with and without a hamstring load of 29 N. Omnibus MANOVA results for seven limbs are reported following exclusion of multivariate outliers both with and without a hamstring load, with Bonferroni-corrected pairwise comparisons underneath. Comparisons are based on landmarks separated by 60° and identified by the midpoint of initial and final caudal joint angles, such that m90° represents the movement from 60° to 120°

	Angle	m85°	m90°	m95°	m100°	m105°
Without hamstring load	V	0.88	0.63	0.54	0.47	1.5
	F	4	2.7	2.2	1.8	3
	p	0.005	0.03	0.06	0.12	0.11
	${\omega }_{p}^{2}$	0.33	0.19	0.14	0.1	0.48
	Intact-CCLx	< 0.001	< 0.001	< 0.001	0.003	0.007
	Intact-MMR	< 0.001	0.002	0.009	0.04	0.02
	Intact-CBLO	0.02	0.02	0.01	0.01	0.008
	CCLx-MMR	0.2	0.8	0.68	0.53	0.52
	CCLx-CBLO	0.02	0.23	0.48	0.87	0.88
	MMR-CBLO	0.16	0.54	0.95	0.75	0.78
With hamstring load	V	1.3	1.32	1.25	1.44	1.36
	F	9.3	11.5	10.1	12.8	10.7
	p	< 0.001	< 0.001	< 0.001	< 0.001	< 0.001
	${\omega }_{p}^{2}$	0.57	0.59	0.56	0.66	0.61
	Intact-CCLx	0.004	0.002	< 0.001	< 0.001	< 0.001
	Intact-MMR	< 0.001	< 0.001	< 0.001	< 0.001	< 0.001
	Intact-CBLO	0.002	0.01	0.04	0.03	0.02
	CCLx-MMR	0.001	< 0.001	< 0.001	< 0.001	< 0.001
	CCLx-CBLO	< 0.001	< 0.001	< 0.001	< 0.001	< 0.001
	MMR-CBLO	< 0.001	< 0.001	< 0.001	< 0.001	< 0.001

V – Pillai’s trace; F – test statistic; *p* – significance; ${\omega }_{p}^{2}$ – partial omega-squared effect size; CCLx – transection of cranial cruciate ligament; MMR – medial meniscal release; CBLO – CORA-based levelling osteotomy

No differences in ICR locations at any midpoint angle were observed after addition of the hamstring load for CCLx, MMR or CBLO (Supplementary Table 4).

### Percentage gliding

Percentage gliding remained stable across joint angles in intact stifles (${\text{{\rm X}}}^{2}$(4) = 6.9, *p* = 0.14, W = 0.25), and for both CCLx and MMR with a hamstring load, although at a higher baseline (${\text{{\rm X}}}^{2}$(4) = 5.9, *p* = 0.2, W = 0.21; ${\text{{\rm X}}}^{2}$(4) = 5.3, *p* = 0.3, W = 0.19). Without a hamstring load, CCLx and MMR exhibited decreasing percentage gliding albeit from a higher baseline (${\text{{\rm X}}}^{2}$(4) = 11.8, *p* = 0.02, W = 0.42; ${\text{{\rm X}}}^{2}$(4) = 12.7, *p* = 0.01, W = 0.45). For CBLO with or without a hamstring load, a gradual increase in percentage gliding was observed (${\text{{\rm X}}}^{2}$(4) = 14.7, *p* = 0.005, W = 0.53; ${\text{{\rm X}}}^{2}$(4) = 16.7, *p* = 0.002, W = 0.60).

**Fig. 2:**
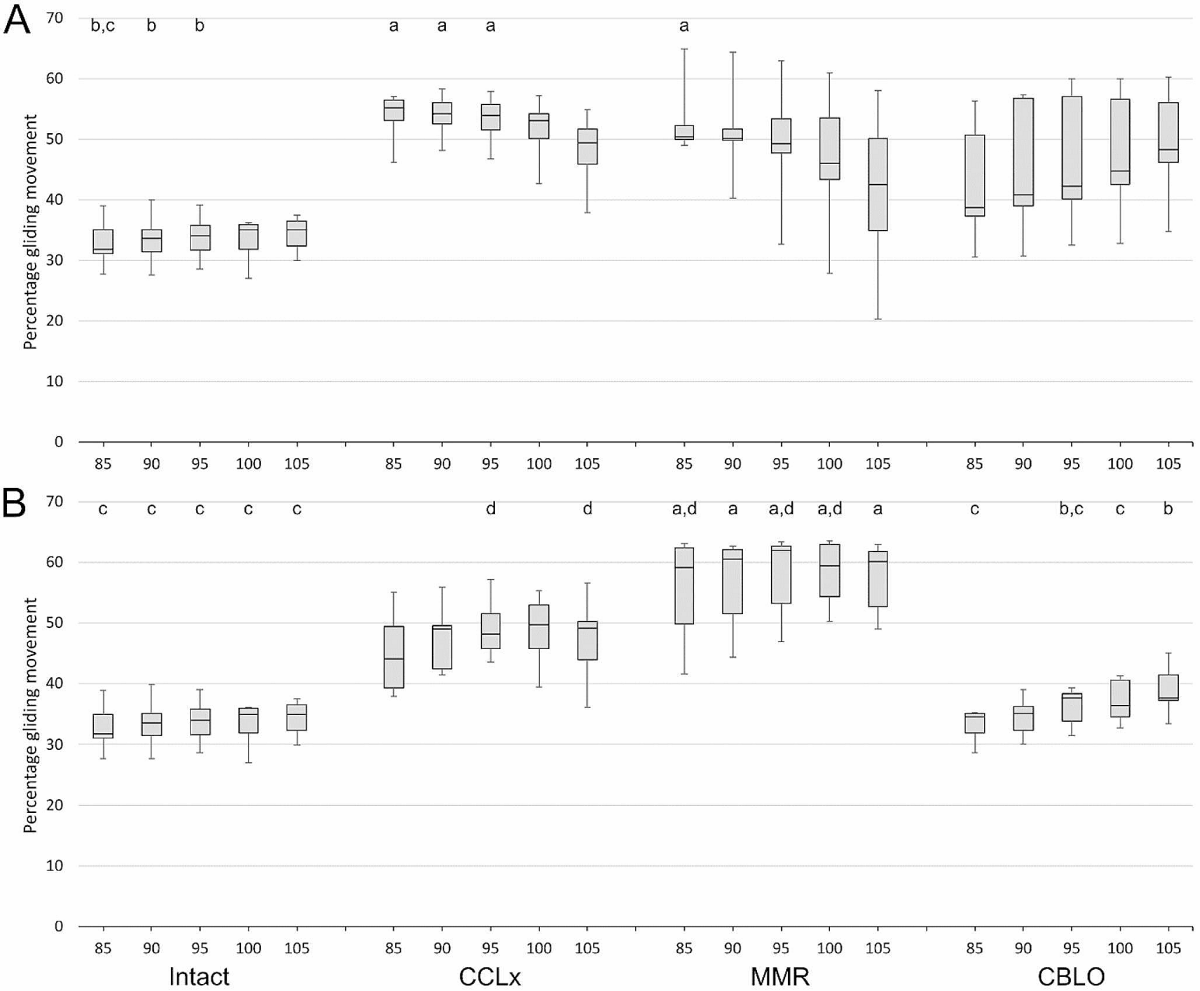
Percentage gliding under varying caudal joint angles, joint stability conditions, and absence (A) or presence (B) of a hamstring load. Box and whisker plots of percentage gliding for the four joint stability conditions (intact, cranial cruciate ligament transection – CCLx, medial meniscal release – MMR, and CORA-based leveling osteotomy – CBLO). The shaded box represents the interquartile range, bisected by the median. The whiskers extend to minimum and maximum values. Numbers represent the angular midpoints between initial and final joint angles for rotations of 60°. Letters indicate significant differences between joint stability conditions at each angle and hamstring load (a – intact, b – CCLx, c – MMR, d – CBLO)

Without a hamstring load, percentage gliding differed significantly between joint stability conditions at angles m85°-m100° (*p* < 0.04, W = 0.40–0.66), with pairwise comparisons showing that MMR, CCLx and CBLO did not differ at any angle, while intact and CCLx differed from m85°-m95°, and intact and MMR at m85° (Supplementary Table 4; Fig. 2A). When a hamstring load was applied, gliding differed significantly at all angles (*p* < 0.001, W = 0.80–0.92). Intact and CCLx values no longer differed at any angle, and intact and MMR values consistently differed, while differences between CBLO and both CCLx and MMR became apparent. Data variability was visually reduced for MMR and CBLO conditions (Fig. 2B).

Direct comparisons of the effect of a hamstring load for each stability condition and angle demonstrated differences for CCLx at m85° and m90°, for MMR and m105°, and CBLO at m85°, m100° and m105°.

## Discussion

Our data suggest that CBLO does not fully restore joint biomechanics, even with a hamstring load. While hamstring loading qualitatively improved ICR path location after CBLO, and reduced path variability based on error ellipse sizes, as well as restoring near normal percentage gliding, significant differences in ICR path compared to intact joints remained. Without hamstring loading, percentage gliding remained qualitatively worse following CBLO compared to the intact joints.

Unlike other osteotomy techniques for managing CCL rupture, mild residual instability is a stated aim of the CBLO procedure, with the goal of preventing loss of compliance in the cranial joint structures due to cranial subluxation of the femoral condyles (Vasquez et al. 2018). This may explain why, despite an acceptable tibial plateau angle following CBLO, the ICR path without hamstring loading remained relatively distant to the intact location. Complete restoration of the ICR path is unlikely to occur following complete CCL rupture or transection, since the path depends on the interaction between the two cruciate ligaments and in intact joints is located at their intersection (O’Connor and Zavatsky 1990). However, it seems likely that achieving an ICR path as close to normal as possible will limit changes in biomechanical function and should prompt clinicians to early intervention in cases with partial CCL rupture, and preservation of functional CCL tissue. The qualitative improvement in ICR path with a hamstring load suggests that postoperative function may be more dependent on muscular balance than the triple tibial osteotomy technique previously tested (Mazdarani et al. 2022, 2023). Abnormal hamstring activity has been reported in dogs affected by CCL rupture (Hayes et al. 2013), and physiotherapy to strengthen these muscles is recognized as a component of postoperative rehabilitation (Kirkby Shaw et al. 2020).

The ICR paths for intact, CCLx, and MMR joints without a hamstring load were qualitatively similar to those reported previously (Mazdarani et al. 2023), increasing confidence in the methodology presented here. Visually, addition of a hamstring load improved femorotibial contact point location placement for CCLx and MMR, consistent with the reduction in percentage gliding noted at lower angles for CCLx but not MMR. In contrast, while femorotibial contact points changed minimally for CBLO with a hamstring load, there was a qualitative improvement in percentage gliding and interlimb variability consistent with the ICR path becoming positioned closer to the intact path. Interestingly, the ICR path following MMR moved further from the intact path with a hamstring load, suggesting that the stabilizing hamstring effect noted for CCLx and CBLO joints might depend on inherent joint stability from the wedge effect of the medial meniscus or reduced cranial tibial thrust due to reduction of the tibial plateau angle (Slocum and Slocum 1993).

The pattern of percentage gliding observed here is consistent with previous reports for triple tibial osteotomy (Mazdarani et al. 2023), and while qualitatively improved by hamstring loading, we were only able to show limited changes statistically. This may reflect a Type II error due to the small sample size coupled with interindividual variability, despite the generally large effect sizes. Sample size was limited by the pre-existing data.

This study has several additional limitations. The joints in the source study were under constant simulated muscle loads but not axially loaded, and human studies have indicated that ICR paths are somewhat dependent on weight-bearing (Hollman et al. 2002). Our results are most consistent with their non-weight bearing scenario. Stifle movement was necessarily reduced to uniplanar movement, whereas in vivo limited rotation and varus-valgus movement also occurs and is exacerbated by CCL incompetency (Korvick et al. 1994; Tinga et al. 2018). This could contribute to interindividual variability in non-intact stifles, as rotation may have affected landmark positions. Three-dimensional analysis to determine the instantaneous (finite) helical axis in humans yields an axis slightly oblique to the mediolateral axis (Bishop et al. 2020). This might also be true in dogs rather than the mediolateral axis assumed here for planar movement: however, our overall trends are likely reliable. The ICR path is highly dependent on starting and ending points, angular displacement, and the presence of purely rotational movement (Chen and Katona 1999). While our methodology addresses some of these issues, and our results appear consistent with previous reports, unstable stifles respond individually rather than consistently to CCLx and MMR, as reflected in the larger error ellipses for these conditions. Whether this individual response was entirely responsible for the multivariate outliers we identified is uncertain. Other contributing factors could include errors in ICR calculation due to landmark error, joint rotation, and reduction to a two-dimensional setting. Percentage gliding used osseous landmarks rather than the articular cartilage surface and given the typical condylar cartilage thickness in dogs of 0.6–0.8 mm (Böttcher et al. 2009) this may have resulted in some overestimation of gliding values. This represents a technique limitation due to the nature of fluoroscopic recordings and will be difficult to avoid in future studies using this modality. Cine MRI scanning during gradual movement might enable better refinement of ICR paths since cartilage surfaces can be visualized (Aleksiev et al. 2022).

In summary, CBLO did not restore the ICR path to the intact location with or without a hamstring load, potentially consistent with CBLO aims of mild residual instability. CBLO did result in percentage gliding characteristics not significantly different to intact joints. Qualitative improvements in ICR path and percentage gliding quantities and variability suggest that hamstring loading positively influences joint biomechanics and that further investigation of this role ex vivo and clinically is warranted.

### Electronic supplementary material

Below is the link to the electronic supplementary material.

Supplementary Material 1

Supplementary Material 2

Supplementary Material 3

Supplementary Material 4

Supplementary Material 5

## Declarations

### Data statement

Supporting data and output from statistical analyses for this study are available online (Miles and Mazdarani 2023).
