# Supplementary material for: Effect of center of rotation of angulation-based levelling osteotomy on instantaneous center of rotation ex vivo
Source: Vet Res Commun. 2024 Jan 29;48(3):1845–51. doi: 10.1007/s11259-024-10314-2 (PMC11147888; doi:10.1007/s11259-024-10314-2)

Supplemental Figure 1: Calculation of percentage rolling and gliding for each mid-rotation position. A best-fit circle was fitted to each condyle, with the center marked (black dot). The femorotibial contact point at mid-rotation is fitted to the best-fit circle (red dot), and the instantaneous center of rotation (ICR) for the rotational movement is marked with a green dot. Two arcs are calculated: $S_{m}$ along the best-fit circle (radius $r_{bf}$), and $S_{f}$ at a radius calculated as $r_{bf}-r_{icr}$. The percentage rolling is calculated using the equation shown: as the ICR approaches the joint surface, $r_{icr}$ approaches zero and $S_{f}$ approaches $S_{m}$, resulting in 100% rolling movement and 0% gliding.


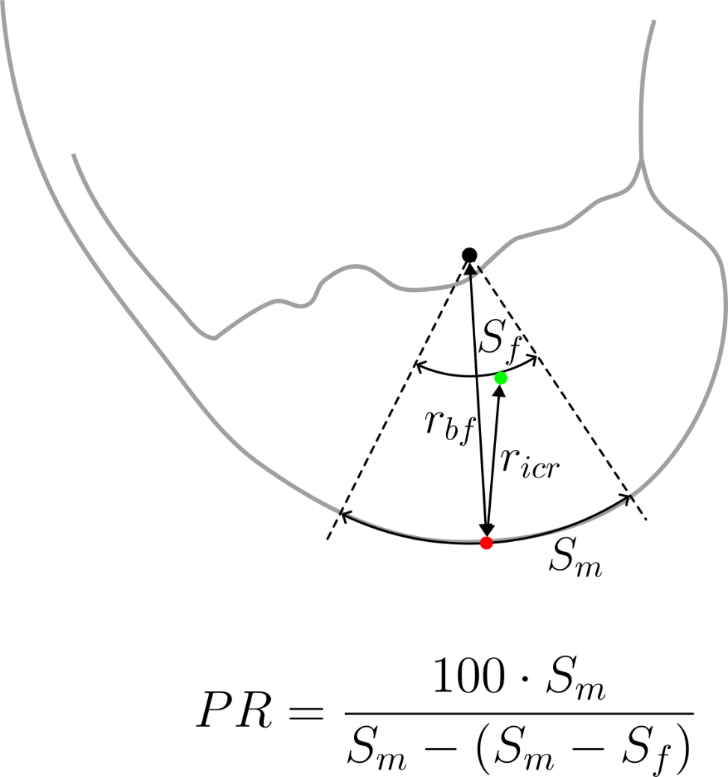

Supplement: Supplementary file 1 — Supplementary Material 1 [file 11259_2024_10314_MOESM1_ESM.docx]
