# Supplementary material for: Effect of center of rotation of angulation-based levelling osteotomy on instantaneous center of rotation ex vivo
Source: Vet Res Commun. 2024 Jan 29;48(3):1845–51. doi: 10.1007/s11259-024-10314-2 (PMC11147888; doi:10.1007/s11259-024-10314-2)
Supplement: Supplementary file 2 — Supplementary Material 2 [file 11259_2024_10314_MOESM2_ESM.docx]

Supplementary Table 1: The effect of caudal joint angle on instantaneous center of rotation location for each joint stability condition both with and without a hamstring load of 29 N. MANOVA results for seven limbs are reported without removal of multivariate outliers.

| Outliers | Joint condition | Hamstring load | V | F | p | $\omega_{p}^{2}$ |
| --- | --- | --- | --- | --- | --- | --- |
| Removed | Intact |  | 0.99 | 5.9 (8) | <0.001 | 0.41 |
|  | CCLx |  | 1.32 | 7.7 (8) | <0.001 | 0.57 |
|  | MMR |  | 0.64 | 1.4 (8) | 0.24 | 0.09 |
|  | CBLO |  | 0.55 | 1.9 (8) | 0.09 | 0.13 |
|  | CCLx | Yes | 0.37 | 1.1 (8) | 0.37 | 0.02 |
|  | MMR | Yes | 0.51 | 1.7 (8) | 0.13 | 0.1 |
|  | CBLO | Yes | 0.92 | 4.3 (8) | <0.001 | 0.35 |
| Included | CCLx |  | 1.02 | 6.3 (8) | <0.001 | 0.43 |
|  | MMR |  | 0.76 | 3.6 (8) | 0.002 | 0.27 |
|  | CBLO |  | 0.48 | 1.9 (8) | 0.09 | 0.11 |
|  | CCLx | Yes | 0.47 | 1.9 (8) | 0.09 | 0.11 |
|  | MMR | Yes | 0.59 | 2.5 (8) | 0.02 | 0.17 |
|  | CBLO | Yes | 0.91 | 5 (8) | <0.001 | 0.36 |

V – Pillai’s trace; F – test statistic with degrees of freedom; p – significance; $\omega_{p}^{2}$ – partial omega-squared effect size; CCLx – transection of cranial cruciate ligament; MMR – medial meniscal release; CBLO – CORA-based levelling osteotomy
