# Supplementary material for: Effect of center of rotation of angulation-based levelling osteotomy on instantaneous center of rotation ex vivo
Source: Vet Res Commun. 2024 Jan 29;48(3):1845–51. doi: 10.1007/s11259-024-10314-2 (PMC11147888; doi:10.1007/s11259-024-10314-2)
Supplement: Supplementary file 3 — Supplementary Material 3 [file 11259_2024_10314_MOESM3_ESM.docx]

Supplementary Table 4: Percentage gliding between joint stability conditions. Pairwise comparisons for seven limbs with Bonferroni-corrected p values between intact joints, following cranial cruciate ligament transection (CCLx), medial meniscal release (MMR), and CORA-based leveling osteotomy (CBLO) both with and without a hamstring load. Comparisons are based on landmarks separated by 60° and referenced by the midpoint (m) of the initial and final caudal joint angles.

|  | Condition pair | m85° | m90° | m95° | m100° | m105° |
| --- | --- | --- | --- | --- | --- | --- |
| Without hamstring load | Intact-CCLx | 0.003 | 0.006 | 0.043 | 0.14 | 0.37 |
|  | Intact-MMR | 0.043 | 0.08 | 0.14 | 0.14 | 0.37 |
|  | Intact-CBLO | 0.59 | 0.23 | 0.23 | 0.08 | 0.08 |
|  | MMR-CCLx | 1 | 1 | 1 | 1 | 1 |
|  | CCL-CBLO | 0.37 | 1 | 1 | 1 | 1 |
|  | MMR-CBLO | 1 | 1 | 1 | 1 | 1 |
| With hamstring load | Intact-CCLx | 0.23 | 0.37 | 0.23 | 0.59 | 0.88 |
|  | Intact-MMR | 0.003 | 0.006 | 0.011 | 0.023 | 0.043 |
|  | Intact-CBLO | 1 | 1 | 1 | 1 | 1 |
|  | MMR-CCLx | 0.88 | 0.88 | 1 | 1 | 1 |
|  | CCL-CBLO | 0.14 | 0.08 | 0.043 | 0.08 | 0.043 |
|  | MMR-CBLO | 0.001 | <0.001 | 0.001 | 0.001 | <0.001 |
