# Supplementary material for: Effect of center of rotation of angulation-based levelling osteotomy on instantaneous center of rotation ex vivo
Source: Vet Res Commun. 2024 Jan 29;48(3):1845–51. doi: 10.1007/s11259-024-10314-2 (PMC11147888; doi:10.1007/s11259-024-10314-2)
Supplement: Supplementary file 4 — Supplementary Material 4 [file 11259_2024_10314_MOESM4_ESM.docx]

Supplementary Table 2: Effect of joint stability condition on instantaneous center of rotation location at tested joint angles with and without a hamstring load. Omnibus MANOVA results for seven limbs are reported following exclusion of multivariate outliers both with and without a hamstring load of 29 N, with Bonferroni-corrected pairwise comparisons underneath. Comparisons are based on landmarks separated by 60° and identified by the midpoint of initial and final caudal joint angles, such that m90° represents the movement from 60° to 120°.

|  | Angle | m85° | m90° | m95° | m100° | m105° |
| --- | --- | --- | --- | --- | --- | --- |
| Without hamstring load | V | 0.85 | 0.63 | 0.54 | 0.47 | 0.54 |
|  | F | 4.5 | 2.7 | 2.2 | 1.8 | 2.2 |
|  | p | 0.002 | 0.03 | 0.06 | 0.12 | 0.06 |
|  | $\omega_{p}^{2}$ | 0.33 | 0.19 | 0.14 | 0.1 | 0.14 |
|  | Intact-CCLx | <0.001 | <0.001 | <0.001 | 0.003 | 0.02 |
|  | Intact-MMR | <0.001 | 0.002 | 0.009 | 0.04 | 0.08 |
|  | Intact-CBLO | 0.04 | 0.02 | 0.01 | 0.01 | 0.007 |
|  | CCLx-MMR | 0.21 | 0.8 | 0.68 | 0.53 | 0.32 |
|  | CCLx-CBLO | 0.04 | 0.23 | 0.48 | 0.87 | 0.69 |
|  | MMR-CBLO | 0.05 | 0.54 | 0.95 | 0.75 | 0.09 |
| With hamstring load | V | 1.34 | 1.32 | 1.25 | 1.23 | 1.16 |
|  | F | 12.1 | 11.5 | 10.1 | 9.5 | 8.3 |
|  | p | <0.001 | <0.001 | <0.001 | <0.001 | <0.001 |
|  | $\omega_{p}^{2}$ | 0.61 | 0.59 | 0.56 | 0.54 | 0.5 |
|  | Intact-CCLx | 0.02 | 0.002 | <0.001 | <0.001 | <0.001 |
|  | Intact-MMR | <0.001 | <0.001 | <0.001 | <0.001 | <0.001 |
|  | Intact-CBLO | 0.003 | 0.01 | 0.04 | 0.08 | 0.08 |
|  | CCLx-MMR | <0.001 | <0.001 | <0.001 | <0.001 | <0.001 |
|  | CCLx-CBLO | <0.001 | <0.001 | <0.001 | <0.001 | <0.001 |
|  | MMR-CBLO | <0.001 | <0.001 | <0.001 | <0.001 | <0.001 |

V – Pillai’s trace; F – test statistic with degrees of freedom; p – significance; $\omega_{p}^{2}$ – partial omega-squared effect size; CCLx – transection of cranial cruciate ligament; MMR – medial meniscal release; CBLO – CORA-based levelling osteotomy
