# Supplementary material for: Effect of center of rotation of angulation-based levelling osteotomy on instantaneous center of rotation ex vivo
Source: Vet Res Commun. 2024 Jan 29;48(3):1845–51. doi: 10.1007/s11259-024-10314-2 (PMC11147888; doi:10.1007/s11259-024-10314-2)
Supplement: Supplementary file 5 — Supplementary Material 5 [file 11259_2024_10314_MOESM5_ESM.docx]

Supplementary Table 3: The effect of hamstring load on instantaneous center of rotation location at each midpoint angle for joint movement. MANOVA results for seven limbs are reported both after removal of multivariate outliers and with all data included.

|  |  | CCLx | | | | MMR | | | | CBLO | | | |
| --- | --- | --- | --- | --- | --- | --- | --- | --- | --- | --- | --- | --- | --- |
|  | m (°) | V | F | p | $\omega_{p}^{2}$ | V | F | p | $\omega_{p}^{2}$ | V | F | p | $\omega_{p}^{2}$ |
| Outliers excluded | 85 | 0.65 | 3.8 (2) | 0.12 | 0.44 | 0.7 | 4.7 (2) | 0.09 | 0.51 | 0.64 | 4.4 (2) | 0.08 | 0.46 |
|  | 90 | 0.59 | 3.6 (2) | 0.11 | 0.39 | 0.31 | 1.1 (2) | 0.4 | 0.02 | 0.64 | 4.4 (2) | 0.08 | 0.46 |
|  | 95 | 0.31 | 1.1 (2) | 0.4 | 0.02 | 0.36 | 1.4 (2) | 0.33 | 0.09 | 0.63 | 4.3 (2) | 0.08 | 0.45 |
|  | 100 | 0.18 | 0.5 (2) | 0.61 | -0.14 | 0.46 | 1.7 (2) | 0.29 | 0.17 | 0.66 | 4.9 (2) | 0.07 | 0.49 |
|  | 105 | 0.05 | 0.1 (2) | 0.93 | -0.43 | 0.61 | 2.3 (2) | 0.25 | 0.3 | 0.64 | 2.6 (2) | 0.22 | 0.35 |
| Outliers retained | 85 | 0.66 | 4.9 (2) | 0.07 | 0.49 | 0.26 | 0.9 (2) | 0.47 | -0.03 | 0.64 | 4.4 (2) | 0.08 | 0.46 |
|  | 90 | 0.59 | 3.6 (2) | 0.11 | 0.39 | 0.31 | 1.1 (2) | 0.4 | 0.02 | 0.64 | 4.4 (2) | 0.08 | 0.46 |
|  | 95 | 0.31 | 1.1 (2) | 0.4 | 0.02 | 0.36 | 1.4 (2) | 0.33 | 0.09 | 0.63 | 4.3 (2) | 0.08 | 0.45 |
|  | 100 | 0.18 | 0.5 (2) | 0.61 | -0.14 | 0.46 | 1.7 (2) | 0.29 | 0.17 | 0.66 | 4.9 (2) | 0.07 | 0.49 |
|  | 105 | 0.27 | 0.9 (2) | 0.45 | -0.03 | 0.46 | 2.1 (2) | 0.22 | 0.22 | 0.69 | 5.6 (2) | 0.05 | 0.53 |

m – midpoint angle for 60° rotation; V – Pillai’s trace; F – test statistic with degrees of freedom; p – significance; $\omega_{p}^{2}$ – partial omega-squared effect size; CCLx – transection of cranial cruciate ligament; MMR – medial meniscal release; CBLO – CORA-based levelling osteotomy
